# Pathologic heterogeneity of lung adenocarcinomas: A novel pathologic index predicts survival

**DOI:** 10.18632/oncotarget.11857

**Published:** 2016-09-06

**Authors:** Geewon Lee, E-Ryung Choi, Ho Yun Lee, Ji Yun Jeong, Joong Hyun Ahn, Seonwoo Kim, Jungmin Bae, Hong Kwan Kim, Yong Soo Choi, Jhingook Kim, Jaeil Zo, Kyung Soo Lee, Young Mog Shim

**Affiliations:** ^1^ Department of Radiology and Center for Imaging Science, Samsung Medical Center, Sungkyunkwan University School of Medicine, Seoul, Korea; ^2^ Department of Radiology and Medical Research Institute, Pusan National University Hospital, Pusan National University School of Medicine, Busan, Korea; ^3^ Department of Pathology, Kyungpook National University Hospital, Kyungpook National University School of Medicine, Daegu, Korea; ^4^ Biostatistics Team, Samsung Biomedical Research Institute, Seoul, Korea; ^5^ Department of Thoracic and Cardiovascular Surgery, Samsung Medical Center, Sungkyunkwan University School of Medicine, Seoul, Korea

**Keywords:** lung adenocarcinoma, heterogeneity, pathology, subtype, survival

## Abstract

Although the most predominant subtype of invasive lung adenocarcinoma has been reported to have clinical significance, a major limitation of this concept is that most tumors are mixed-subtype. Therefore, we aimed to determine the individual prognostic significance of each subtype and also attempted to establish a pathologic index that reflects the pathologic subtypes and overall heterogeneity of lung adenocarcinomas and evaluated its prognostic significance. The individual prognostic impact of each subtype was assessed from the development cohort using the disease-free survival (DFS) curve of a previous large-scale study. Hazard ratios (HRs) from the development cohort were 1, 1.025, 1.059, 1.495, and 1.160 for the lepidic, acinar, papillary, micropapillary, and solid pattern subtype, respectively. Based on the calculated HR of each subtype, four indices representing pathologic heterogeneity were developed. The first and second indices were defined as the sum of the proportions of each subtype multiplied by their HRs, with the addition of either entropy or Gini coefficient, respectively. The third index was calculated as the sum of all subtype percentages multiplied by their HRs. To emphasize heterogeneity, the fourth index was defined as the simple arithmetic sum of the scores of the subtypes multiplied by their HRs. Each subtype was assigned a score of 0 if the subtype was absent and a score of 1 if the subtype was present in a binary fashion. We applied these four pathologic indices to a validation group of 148 patients with comprehensive histologic subtyping for completely resected lung adenocarcinomas. DFS curves were plotted and predictive ability of each pathologic index was evaluated. Among the four pathologic indices, only pathologic index 3 enabled significant patient stratification in the validation cohort according to DFS (*P* = 0.004) and showed the highest Harrell's C index of 0.691 of all four pathologic indices. In conclusion, we estimated the HR of each subtype and generated four pathologic indices that reflect heterogeneity. One of these, index 3, the pathologic heterogeneity index based on the sum of all subtype percentages multiplied by their HR, possesses good prognostic ability for predicting survival in patients with lung adenocarcinoma.

## INTRODUCTION

In 2011, a new classification system for lung adenocarcinoma was published by the International Association for the Study of Lung Cancer (IASLC), American Thoracic Society (ATS), and European Respiratory Society (ERS) [1]. This new classification system introduced five distinct architectural subtype patterns of invasive lung adenocarcinoma: lepidic, acinar, solid, papillary, and micropapillary. Since the publication of this classification, many studies have investigated its clinical significance and have validated the most predominant subtype as a promising index for patient stratification into several prognostic groups [2–4]. However, a major limitation of the most predominant subtype concept is that only about 20% of all lung adenocarcinomas are pure-subtype, e.g. composed of a single subtype [5]. Most lung adenocarcinomas are mixed-subtype, e.g. composed of 2 or more different architectural subtypes, and in one study [6], mixed-subtype adenocarcinoma accounted for 94% of all lung adenocarcinomas.

Even with the same most predominant subtype, tumors may show diverse biologic behaviors. For example, the following three tumors all have acinar predominant lung adenocarcinoma: (1) lung adenocarcinoma with 60% acinar subtype + 40% lepidic subtype; (2) lung adenocarcinoma with 60% acinar subtype + 40% solid subtype; and (3) lung adenocarcinoma with 60% acinar subtype + 40% papillary subtype. However, the second most predominant subtypes of these tumors have different tumor aggressiveness, thus raising the question of whether these three tumors all have identical outcomes. Specific subtypes have been proposed to contribute to the total biologic behavior of a tumor and to influence prognosis in mixed-subtype adenocarcinomas [7, 8]. Therefore, the most predominant subtype is not completely representative of the whole tumor. Moreover, applying the most predominant subtype concept means that other minor yet important components may be excluded from consideration. In this context, we aimed to determine the relative prognostic significance of each subtype from a development cohort using data from a large study group [9]. Furthermore, we attempted to establish a pathologic index based on the relative prognostic significance of each subtype that accurately reflects the pathologic subtypes and overall heterogeneity of lung adenocarcinomas. We also evaluated the prognostic significance of this index in a validation cohort.

## RESULTS

### Prognostic significance of each subtype From the development cohort

**Figure 1:**
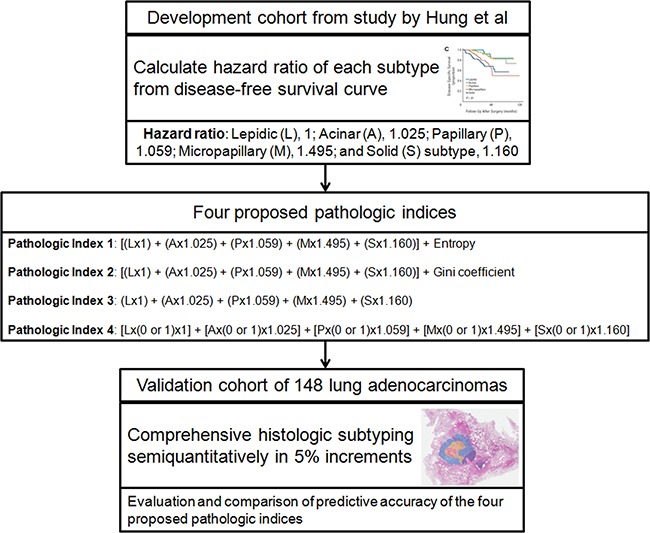
Study design flow chart In the first step, the hazard ratio of each subtype was calculated from the disease-free survival curve of the development cohort, which was obtained from the study by Hung et al [9]. Next, four proposed pathologic indices were generated. Finally, the predictive accuracies of the four proposed pathologic indices were compared using the validation cohort of 148 patients.

The hazard ratios (HRs) calculated from the development cohort were 1, 1.025, 1.059, 1.495, and 1.160 for the lepidic, acinar, papillary, micropapillary, and solid subtypes, respectively (Figure 1).

### Demographics of the validation cohort

The clinical and pathologic characteristics of the validation cohort are listed in Table 1. The most frequently observed predominant subtype was acinar (94 patients; 63.5%), followed by lepidic (41 patients; 27.7%), papillary (7 patients; 4.7%), solid (4 patients; 2.7%), and micropapillary (2 patients; 1.4%). Eighty-two (55.4%) patients had internal scar tissue, with the scar tissue areas ranging from 5-70%, while 66 (44.6%) patients did not exhibit any internal scar tissue in their tumor specimens. In terms of tumor heterogeneity, 32 (21.6%) patients had pure-subtype tumors and 116 (78.4%) patients had mixed-subtype tumors. Among these 116 patients, 95 (64.2%), 20 (13.5%), and 1 (0.7%) patient had tumors composed of two, three, and four subtypes, respectively. No patient exhibited all five subtypes in any tumor specimen.

**Table 1:** Clinical and pathologic characteristics of the validation cohort

Characteristic	No. of patients (N=148)	%^*^
**Age (mean ± SD) (years)**	59 ± 8.6	
**Sex**		
Male	66	44.6
Female	82	55.4
**Tumor size (mean ± SD) (mm)**	22 ± 8	
**Pathologic stage**		
Ia	95	64.2
Ib	32	21.6
IIa	4	2.7
IIb	10	6.8
IIIa	7	4.7
**T Status**		
pT1	102	68.9
pT2	41	27.7
pT3	5	3.4
**N Status**		
pN0	130	87.8
pN1	11	7.4
pN2	7	4.7
**Operation type**		
Wedge resection/segmentectomy	16	10.8
Lobectomy	127	85.8
Bilobectomy	3	2.0
Pneumonectomy	2	1.4
**Predominant subtype**		
Lepidic	41	27.7
Acinar	94	63.5
Papillary	7	4.7
Micropapillary	2	1.4
Solid	4	2.7
**Scar tissue**		
Present	82	55.4
Absent	66	44.6
**No. of subtypes comprising the tumor**		
1	32	21.6
2	95	64.2
3	20	13.5
4	1	0.7

SD = Standard deviation,

* Due to rounding, percentages do not necessarily add up to 100.

The relationships between the predominant subtype and the presence of other nonpredominant subtypes in the validation cohort are shown in Table 2. Among the five subtypes, acinar (132 patients; 89.2%) was observed in the majority of patients, followed by lepidic (99 patients; 66.9%), solid (24 patients; 16.2%), papillary (17 patients; 11.5%), and micropapillary (16 patients; 10.8%). The relationships between the predominant subtype and the number of subtypes comprising each tumor in the validation cohort are shown in Table 3.

**Table 2:** Relationships between the predominant subtype and the presence of other subtypes in the validation cohort

Predominant subtype	Number of patients with the subtype present				
	Lepidic	Acinar	Papillary	Micropapillary	Solid
**Lepidic (n=41)**	41	29	0	0	1
**Acinar (n=94)**	53	94	9	13	18
**Papillary (n=7)**	4	6	7	1	1
**Micropapillary (n=2)**	0	1	1	2	0
**Solid (n=4)**	1	2	0	0	4
**Total (%)**	99 (66.9)	132 (89.2)	17 (11.5)	16 (10.8)	24 (16.2)

**Table 3:** Relationships between the predominant subtype and the number of subtypes comprising the tumor in the validation cohort

Predominant subtype	Number of subtypes comprising the tumor				
	1	2	3	4	5
**Lepidic (n=41)**	12 (29)	29 (71)	0 (0)	0 (0)	0 (0)
**Acinar (n=94)**	19 (20)	59 (63)	15 (16)	1 (1)	0 (0)
**Papillary (n=7)**	0 (0)	2 (29)	5 (71)	0 (0)	0 (0)
**Micropapillary (n=2)**	0 (0)	2 (100)	0 (0)	0 (0)	0 (0)
**Solid (n=4)**	1 (25)	3 (75)	0 (0)	0 (0)	0 (0)

Numbers in parentheses are percentages.

The median follow-up period of the validation cohort was 48.2 ± 15.1 months (range, 9.9 to 90.8 months). Among the 148 patients, 32 patients demonstrated disease recurrence during follow-up. Median disease-free survival (DFS) was 83.1 months. Only 2 patients died during follow-up, of which one died from recurrent lung cancer, and the other died without recurrence.

### Association between proposed pathologic indices and survival in the validation cohort

**Figure 2:**
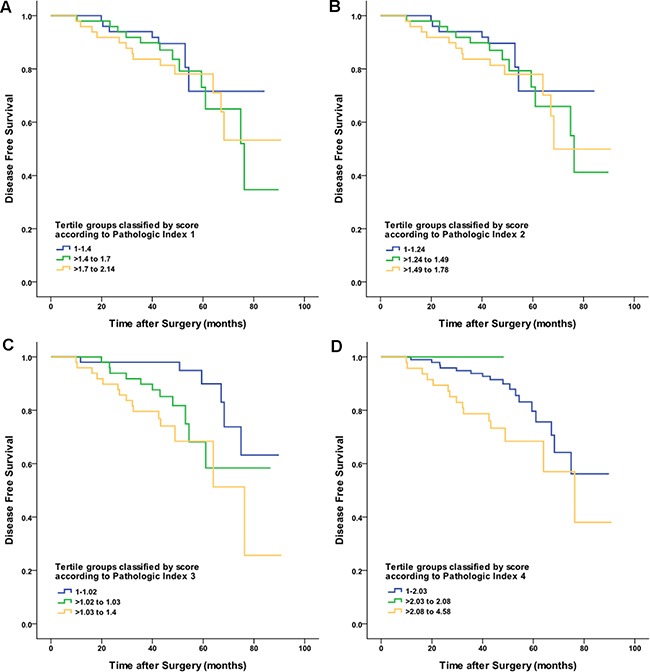
Disease-free survival curves for the validation cohort tertiles of the four proposed pathologic indices Patients were stratified according to the following score: **A.** pathologic index 1; **B.** pathologic index 2; **C.** pathologic index 3; and **D.** pathologic index 4.

Using the calculated scores, the validation cohort was divided into tertile groups consisting of 50, 49, and 49 patients for each pathologic index. DFS curves for the validation cohort tertiles of the four proposed pathologic indices are shown in Figure 2. Among tertile groups, DFS curves were different with statistical significance for only pathologic index 3 (log-rank *P* value = 0.004). In contrast, the other three pathologic indices were not able to significantly stratify patients in the validation cohort according to their DFS.

The associations between each pathologic index and the clinical outcomes in the validation cohort are demonstrated in Table 4. Among the four proposed pathologic indices, the Harrell's C index of pathologic index 3 was the highest (0.691; 95% Confidence interval: 0.633-0.749). In our study, the incorporation of commonly used measurements of heterogeneity, such as entropy and the Gini coefficient, did not result in better stratification of patient survival (Harrell's C index of 0.572 and 0.575 for pathologic indices 1 and 2, respectively).

**Table 4:** Associations between proposed pathologic indices and clinical outcomes in the validation cohort

Pathologic index	Score	Hazard ratio (95% CI)	*P*-value	Harrell's C index	Concordance probability estimate
**1**	1-1.4	1	0.589	0.572 (0.514-0.630)	0.558 (0.507-0.610)
	>1.4 to 1.7	1.525 (0.597-3.893)			
	>1.7 to 2.14	1.543 (0.610-3.905)			
**2**	1-1.24	1	0.563	0.575 (0.517-0.633)	0.555 (0.502-0.609)
	>1.24 to 1.49	1.475 (0.576-3.776)			
	>1.49 to 1.78	1.618 (0.641-4.086)			
**3**	1-1.02	1	0.004	0.691 (0.633-0.749)	0.542 (0.520-0.564)
	>1.02 to 1.03	2.899 (1.056-7.959)			
	>1.03 to 1.4	4.489 (1.713-11.764)			
**4**	1-2.03	1	0.051	0.642 (0.587-0.697)	0.573 (0.531-0.615)
	>2.03 to 2.08	NA*			
	>2.08 to 4.58	2.207 (1.100-4.428)			

CI = Confidence interval. *NA, not applicable due to the lack of events in the tertile.

*P*-value for each pathologic index was calculated using the log-rank test among tertile groups for survival difference.

Concordance probability estimate was calculated according to Gonen and Heller Concordance Index for Cox models.

### Multivariate analysis of proposed pathologic indices and clinical parameters

Multivariate analysis (Table 5) identified TNM stage (*P* < 0.001) and tertile group according to proposed pathologic index 3 (*P* = 0.017) as independent prognostic factors. Age, sex, and other proposed pathologic indices were not identified as predictive factors.

**Table 5:** Multivariate analysis of proposed pathologic index 3 and clinical parameters for DFS

Variable	Hazard ratio (95% CI)	*P*-value
**Age**	0.984 (0.947-1.023)	0.428
**Sex**	0.855 (0.397-1.841)	0.689
**TNM Stage**	1.893 (1.403-2.554)	^*^<0.001
**Tertile group according to proposed pathologic index 1**	1.366 (0.203-9.205)	0.748
**Tertile group according to proposed pathologic index 2**	0.878 (0.132-5.816)	0.893
**Tertile group according to proposed pathologic index 3**	3.012 (1.223-7.418)	^*^0.017
**Tertile group according to proposed pathologic index 4**	0.508 (0.230-1.126)	0.095

* Statistically significant at *P* < 0.05.

### Performance comparison of proposed pathologic indices and predominant subtype

**Figure 3:**
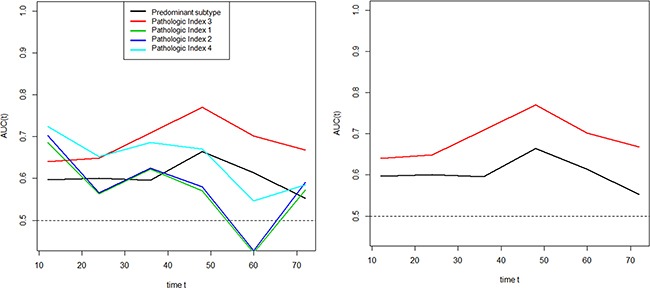
Area under time-dependent ROC curves (AUC) according to the predominant subtype and proposed pathologic indices Proposed pathologic indices 3, 4, and the predominant subtype had AUC values over 0.5 (left). AUC values of proposed pathologic index 3 were constantly higher than the predominant subtype regardless of time.

Time-dependent receiver operating characteristics (ROC) curve shows that area under the ROC curve (AUC) values were over 0.5 for proposed pathologic indices 3, 4, and the predominant subtype (Figure 3). In particular, AUC values of proposed pathologic index 3 are constantly over the predominant subtype regardless of time.

## DISCUSSION

Consistent with a number of other studies, Hung et al [9] showed that the most predominant subtype is a stage-independent predictor of DFS. Here we used the large study dataset from Hung et al [9] as a development cohort and assessed the HR of each subtype. Using the HR of each subtype calculated from the development cohort, we generated four pathologic indices that we evaluated in our validation cohort. In a previous effort to create a pattern score, Warth et al [3] assigned 1 to 5 points to each subtype depending on its impact on survival. Although assigning points has the advantage of simplicity, it has the disadvantage of overlooking the absolute prognostic impact of each subtype. Furthermore, this approach did not identify any scoring system that added prognostic value to the predominant subtype concept [3]. Thus, our study is unique in that it was the first to calculate the relative HRs of each subtype and use these HRs to generate pathologic indices. One of these indices (pathologic index 3), hereafter referred to as the “pathologic heterogeneity index”, showed predictive value along with the TNM stage. Furthermore, our pathologic heterogeneity index demonstrated prognostic superiority over the currently established predominant subtype. To the best of our knowledge, no previous study has successfully developed an index that reflects the pathologic subtypes and overall heterogeneity of lung adenocarcinomas and demonstrates predictive value for survival.

Similar to the results of previous reports [5, 6], 21.6% of patients in the validation cohort had pure-subtype tumors, whereas the remaining 78.4% of patients had mixed-subtype tumors, reflecting the fact that a single most predominant subtype is not entirely representative of a large proportion of the tumors. Notably, acinar and papillary-predominant tumors, which constitute a major proportion of lung adenocarcinomas, have been reported to be highly heterogeneous in terms of tumor behavior compared with other subtype tumors, and can range from mild to aggressive tumors [3, 10]. Thus, acinar and papillary-predominant tumors have been referred to as the intermediate-grade group [2, 4, 10, 11]. Our results concur with those of previous studies [2, 4, 11] in that acinar and papillary subtypes had similar intermediate prognostic impacts. Specifically, the HRs for acinar and papillary subtypes were 1.025 and 1.059, respectively. We also found that acinar and papillary-predominant tumors were more often mixed-subtype tumors (80% and 100%, respectively) compared with lepidic-predominant tumors (71%). The larger proportion of mixed-subtype tumors might explain the inconsistent survival rates of acinar and papillary-predominant tumors, which have even been observed in large patient cohorts. Warth et al [3] reported that papillary-predominant tumors were more aggressive than solid-predominant tumors, whereas Yoshizawa [4] and Russell [2] found that solid-predominant tumors were more aggressive than papillary and acinar-predominant tumors. These results indicate that the type and proportion of nonpredominant subtypes may contribute more to the overall heterogeneity in tumor survival than was previously expected [2–4]. In this context, the pathologic heterogeneity index from our study may potentially complement further stratification and redistribution of patient survival among this heterogeneous tumor group with intermediate-grade, mixed-subtype lung adenocarcinomas.

As shown in Table 2, only 4 (2.7%) and 2 (1.4%) patients had solid and micropapillary predominant lung adenocarcinomas, respectively. In contrast, 24 (16.2%) and 17 (11.5%) patients had the presence of the solid or micropapillary subtype in the entire tumor specimen, respectively. This discrepancy indicates that observing the most predominant subtype may inadvertently bias the clinician; thus, minor components with favorable or unfavorable effects on tumor behavior may be overlooked. Previous studies have shown that even the mere presence of the solid or micropapillary subtype is associated with worse survival [1, 12–14]. For example, no significant difference was observed between micropapillary predominant lung adenocarcinomas and non-micropapillary predominant adenocarcinomas with a micropapillary component of greater than or equal to 5% in terms of TNM stage and lymphovascular invasion [14]. Also, limited resection of small lung cancers with micropapillary components greater than or equal to 5% was associated with a significantly greater risk of recurrence [7]. Thus, even a small micropapillary subtype component of 5% may be sufficient to cause lymphovascular invasion and nodal metastasis [7, 14, 15]. In addition, Cha et al [12] demonstrated that the solid subtype contributed to prognosis when it coexisted with the micropapillary subtype.

Sica et al [11] proposed a three-tiered grading system for lung adenocarcinomas: bronchioloalveolar pattern, a discontinued term for lepidic, as grade 1; acinar and papillary as grade 2; and solid and micropapillary as grade 3. Using this grading system, Sica et al [11] showed that the sum of the two most predominant grades classified patients better than the single most predominant subtype. However, this approach cannot distinguish between a tumor with 90% lepidic subtype + 10% micropapillary subtype and a tumor with 10% lepidic subtype + 90% micropapillary subtype. In other words, the grading system proposed by Sica et al [11] did not consider the amount of each subtype; thus, additional measurements are needed to incorporate this information into the scoring system. In this context, our proposed pathologic heterogeneity index is more informative because it takes into consideration the characteristic and proportion of all subtypes. Furthermore, a general limitation of previous pathologic grading systems that grouped subtypes with similar prognoses together is that this type of categorization itself loses the ability to discriminate between the different subtypes [4, 11, 16]. Our data suggest that all subtypes contribute to the whole tumor behavior and overall prognosis; furthermore, both the quantity and quality of each subtype are important.

Approximately 55% of the patients in this study had scar tissue within their tumors. The prognostic importance of scar tissue within lung adenocarcinomas has been investigated in several studies. Maeshima et al. [17] proposed a modified scar grading system that could distinguish invasive lung cancers with low malignant potential from actively invasive lung cancers. However, in a study by Lee et al [18], pathologic central fibrosis was not a risk factor for predicting tumor recurrence. In terms of comprehensive histologic subtyping in lung adenocarcinomas, it appears to be essential to distinguish scar tissue from tumor tissue.

Our study had several limitations. First, we acknowledge that visual estimation has its limitations considering accuracy in the quantitative histologic subtyping of lung adenocarcinomas. To minimize controversy, the two experienced pathologists participated in joint interpretation under a multihead microscope until consensus was achieved. Second, all patients in the validation cohort were from a single institution. Thus, our pathologic index needs to be validated in different regions to determine if it has broad prognostic value. Despite this limitation, the proportions of patients with mixed-subtype tumors were similar to those of previous studies. Third, we excluded patients with variant subtypes including mucinous pattern and signet ring cell pattern. However, we chose to exclude these subtypes because reports regarding the survival of mucinous and signet ring cell lung adenocarcinomas are limited [3, 19]. Further investigations are needed to explore the prognostic significance of these infrequent patterns. Fourth, although cytologic atypia and mitotic counts have been reported to have prognostic value in some studies, we did not investigate these features [20–22]. The current IASLC/ATS/ERS classification recommends assessment of all histologic subtypes semiquantitatively in 5% increments and we designed our scoring system to be based on already available information [1]. Finally, due to the retrospective nature of our study, we lacked potentially relevant genetic information for the majority of our patients, such as EGFR mutations, KRAS mutations, and ALK rearrangements.

In conclusion, here we determined the relative prognostic impact of each subtype in the development cohort and identified a pathologic heterogeneity index with the ability to predict survival in the validation cohort. We anticipate that our proposed pathologic heterogeneity index may complement more precise prognosis stratification and selection of appropriate therapeutic strategies for patients with lung adenocarcinoma. Furthermore, it would be of great interest if our proposed pathologic heterogeneity index were to be validated in future studies.

## MATERIALS AND METHODS

The institutional review board (IRB no. SMC 2011-09-083) of Samsung Medical Center approved this retrospective study and waived the requirement for informed consent.

### Development cohort for estimating individual prognostic significance of each subtype

To estimate the relative prognostic significance of each subtype, a large Taiwanese patient series recently published in 2014 by Hung et al [9] was used as the development cohort. Thus, the development cohort consisted of 573 patients with a median follow-up period of 47.0 months (Supplementary Table S1). All patients had a diagnosis of invasive lung adenocarcinoma that was confirmed by pathologic assessment of the resected specimen. For each tumor, comprehensive histologic subtyping results were available with the percentage of each histologic subtype in 5% increments. In this study [9], the IASLC/ATS/ERS classification system was found to have significant prognostic and predictive value in terms of both survival and recurrence. Using the disease-specific survival curve presented in that study (Supplementary Figure S1) [9], the hazard ratio (HR) of each subtype was calculated as previously reported (Figure 1) [23].

### Four proposed pathologic indices

Based on the relative prognostic significance of each subtype, four individual pathologic indices were developed (Figure 1). The first pathologic index (pathologic index 1) was defined as the sum of the proportions of each subtype multiplied by their HRs, with the addition of entropy. Entropy was calculated as: Entropy = −Σ(p*_i_*)ln(p*_i_*), where (p*_i_*) represents the proportion of subtypes in the tumor [sum of all (p*_i_*) = 1] [24]. For tumors with a single subtype, the logarithmic score is thus 0 (i.e. log 1=0), whereas for tumors of two or more subtypes, the entropy increases. Thus, a higher entropy score represents increased heterogeneity of the tumor. The second pathologic index (pathologic index 2) was defined as the sum of the proportions of each subtype multiplied by their HRs, with the addition of the Gini coefficient. The Gini coefficient was calculated using the following equation: Gini coefficient = 1-Σ(*p_i_*^2^). A Gini coefficient of 0 represents a tumor of pure pathologic type (perfect equality), whereas a Gini coefficient of 0.8 corresponds to a tumor with all five subtypes present at equal frequencies of 0.2 (perfect inequality). The first and second pathologic indices were designed to take into consideration the extent and characteristic of each subtype comprising the tumor, along with the addition of standard methods commonly used to measure heterogeneity [24]. Using similar methodology, the third pathologic index (pathologic index 3) was calculated as the sum of all subtype percentages multiplied by their HRs. To emphasize heterogeneity, the fourth pathologic index (pathologic index 4) was defined as the simple arithmetic sum of the scores of the subtypes multiplied by their HRs. Each subtype was assigned a score of 0 if the subtype was absent and a score of 1 if the subtype was present. Thus, each subtype contributed to the score of the fourth pathologic index in a binary fashion.

### Validation cohort for evaluation and comparison of proposed pathologic indices

For external validation of the proposed pathologic indices, we prepared an independent patient database. Between July 2003 and December 2007, 176 patients who underwent complete surgical resection for lung adenocarcinoma without neoadjuvant therapy at Samsung Medical Center (Seoul, Korea) were identified from a thoracic surgical database. Among these patients, 28 were excluded because of the following pathological factors: variant subtypes, including invasive mucinous adenocarcinoma and signet ring cell adenocarcinoma (n=13); insufficient pathologic slides for evaluation of the whole tumor (n=8); limited tumor evaluation due to combined extensive infarction or inflammation (n=6); and adenocarcinoma combined with spindle cell carcinoma (n=1). Thus, 148 patients (66 males, 82 females; mean age, 59 years) were included in the validation cohort for independent external validation. All clinical information was obtained from the patient electronic medical records.

Whole tumor tissue samples from the entire tumor specimen were placed on a slide. The tissue samples were taken at intervals of approximately 10 mm. Two experienced lung pathologists reviewed a minimum of three hematoxylin and eosin-stained slides per patient. Discrepancies were resolved by consensus in a joint review using a multihead microscope. Comprehensive histologic subtyping was performed semiquantitatively in 5% increments, according to the current IASLC/ATS/ERS lung adenocarcinoma classification system [1]. In tumors with internal scar tissue, defined as areas of fibroblastic focus associated with collagen or hyalinized tissue, the scar tissue area was excluded from the whole tumor measurement. Therefore, disregarding scar tissue, the proportions of each subtype added up to a total of 100% subtype components per tumor (Supplementary Figure S2). If several subtypes were present in a tumor, the subtype that constituted the greatest percentage of the tumor was defined as the most predominant subtype. Using the validation cohort of 148 patients with comprehensive histologic subtyping for completely resected lung adenocarcinomas, all four proposed pathologic indices were evaluated and compared in terms of their abilities to predict survival.

### Statistical analysis

To estimate the hazard ratio for each subtype, we have used the published survival curves [9] (Supplementary Figure S1) and followed the four steps, for each non-overlapping time interval, calculating the number alive (events) and at risk, and estimating the log hazard ratio with its variance, and finally combining log hazard ratios [23].

DFS was defined as the time interval from surgical resection to the documentation of recurrence, including locoregional and nodal metastasis. Patients known to be disease-free were censored at the last follow-up. Patients were divided into three groups of 50, 49, and 49 patients, respectively, according to their scores for each pathologic index. DFS curves were plotted by the Kaplan-Meier method and the curves of the tertile groups were compared with the log-rank test. Associations between each pathologic index and DFS were evaluated using a Cox proportional hazards model. In addition, the predictive accuracy of each proposed pathologic index was measured by computing Harrell's c-index [25]. Concordance probability estimate (CPE) was measured according to the Gonen and Heller Concordance index for Cox models [26]. In addition, multivariate Cox regression analysis was performed to identify variables with prognostic value. To compare the performance of proposed pathologic indices and the predominant subtype, time-dependent ROC curve estimation analysis was performed for comparing AUC of multiple indices measured on the same data [27].

All statistical analyses were performed using SPSS for Windows (version 18.0; SPSS, Chicago, IL, USA) and R (version 3.2.0; R Foundation for Statistical Computing; Vienna, Austria; http://www.R-project.org/). *P* values < 0.05 were taken to indicate statistical significance.

## SUPPLEMENTARY FIGURES AND TABLES




