# Supplementary material for: Pathologic heterogeneity of lung adenocarcinomas: A novel pathologic index predicts survival
Source: Oncotarget. 2016 Sep 6;7(43):70353–63. doi: 10.18632/oncotarget.11857 (PMC5342557; doi:10.18632/oncotarget.11857)
Supplement: Supplementary file 1 [file oncotarget-07-70353-s001.pdf]

Pathologic heterogeneity of lung adenocarcinomas: A novel pathologic index predicts survival

SUPPLEMENTARY FIGURES AND TABLES

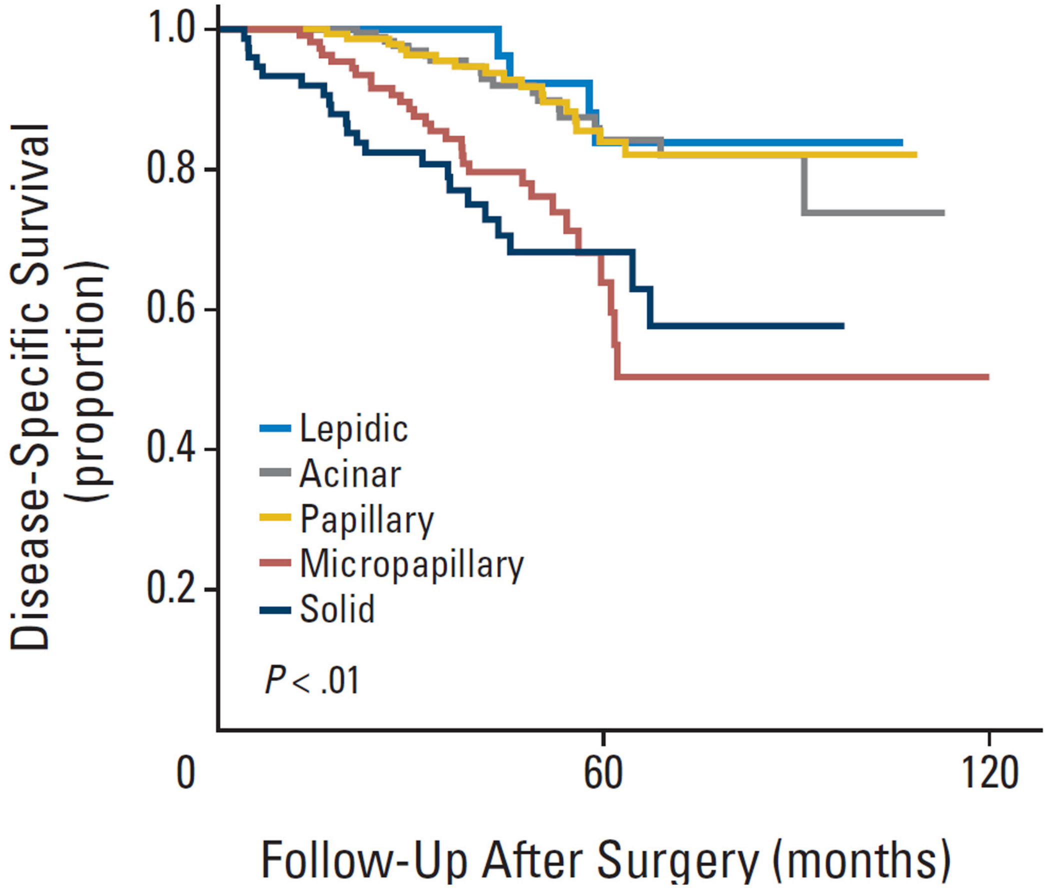

Supplementary Figure S1: Disease-specific survival curve according to all histologic predominant patterns from a previous publication by Hung et al [9].

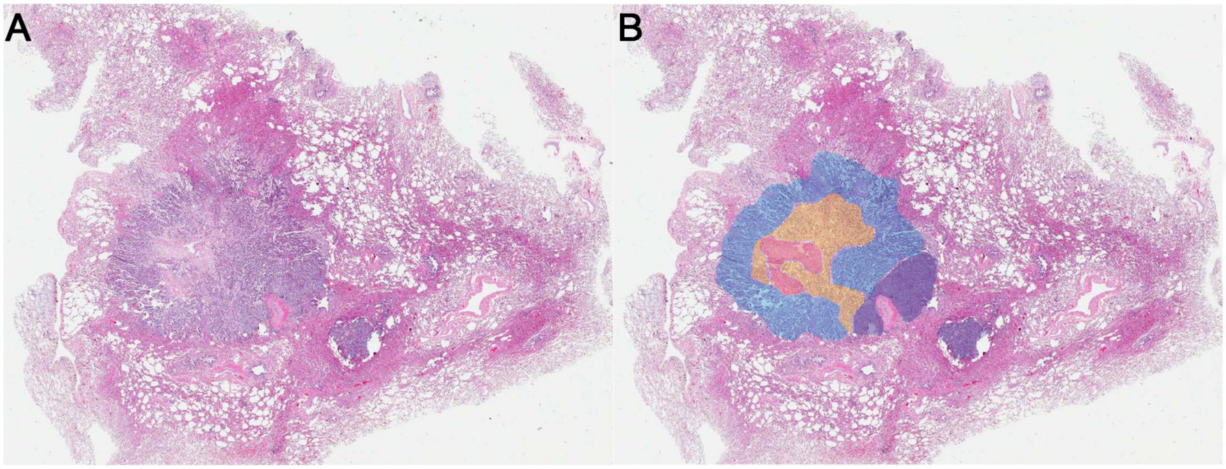

Supplementary Figure S2: Microphotograph demonstrates an example of comprehensive semiquantitative histologic subtyping with elimination of the scar tissue in the validation cohort. **A.** Photomicrograph shows central scar tissue with surrounding areas of tumor cells (hematoxylin-eosin stain). **B.** Schema of tumor cells shows the various components of central scar tissue (red), acinar (yellow), papillary (blue), and solid (purple) subtypes.

Supplementary Table S1: Clinical and pathologic characteristics of the development cohort

Characteristic	No. of Patients (N=573)	%
**Age (mean ± SD) (y)**	63.8 ± 11.6	
**Sex**		
Male	305	53.2
Female	268	46.8
**Tumor size (mean ± SD) (mm)**	28 ± 14	
**Pathologic stage**		
Ia	142	24.8
Ib	232	40.5
IIa	57	9.9
IIb	24	4.2
IIIa	111	19.4
IIIb	7	1.2
**T Status**		
pT1	166	29.0
pT2	349	60.9
pT3	38	6.6
pT4	20	3.5
**N Status**		
pN0	413	72.1
pN1	60	10.5
pN2	100	17.4
**Predominant subtype**		
Lepidic	35	6.1
Acinar	193	33.7
Papillary	155	27.1
Micropapillary	112	19.5
Solid	78	13.6
**No. of subtypes comprising the tumor**		
1	66	11.5
2	251	43.8
3	205	35.8
4	47	8.2
5	4	0.7

SD = Standard deviation

Supplementary Table S2: Number of patterns in each tertile group according to each proposed pathologic index

	No. of patterns	1st tertile				2nd tertile				3rd tertile				P value
		1	2	3	4	1	2	3	4	1	2	3	4	
Pathologic Index	1	30 (60)	20 (40)	0 (0)	0 (0)	0 (0)	46 (94)	3 (6)	0 (0)	2 (4)	29 (59)	17 (35)	1 (2)	<0.001
	2	30 (60)	19 (38)	1 (2)	0 (0)	0 (0)	45 (92)	4 (8)	0 (0)	2 (4)	31 (63)	15 (31)	1 (2)	<0.001
	3	13 (26)	37 (77)	0 (0)	0 (0)	18 (37)	27 (55)	4 (8)	0 (0)	1 (2)	31 (63)	16 (33)	1 (2)	<0.001
	4	32 (33)	65 (67)	0 (0)	0 (0)	0 (0)	4 (100)	0 (0)	0 (0)	0 (0)	26 (55)	20 (43)	1 (2)	<0.001

*Numbers in parentheses are percentages.

Supplementary Table S3: Distribution of the predominant subtype in each tertile group according to each proposed pathologic index.

See Supplementary File 1
