# Supplementary material for: Pathologic heterogeneity of lung adenocarcinomas: A novel pathologic index predicts survival
Source: Oncotarget. 2016 Sep 6;7(43):70353–63. doi: 10.18632/oncotarget.11857 (PMC5342557; doi:10.18632/oncotarget.11857)
Supplement: Supplementary file 2 [file oncotarget-07-70353-s002.docx]

**Supplemental Table 3.** Distribution of the predominant subtype in each tertile group according to each proposed pathologic index.

| Pathologic index |  | 1^st^ tertile | | | | | 2^nd^ tertile | | | | | 3^rd^ tertile | | | | | P value |
| --- | --- | --- | --- | --- | --- | --- | --- | --- | --- | --- | --- | --- | --- | --- | --- | --- | --- |
|  | Predominant subtype | Lep | Aci | Pap | MP | Sol | Lep | Aci | Pap | MP | Sol | Lep | Aci | Pap | MP | Sol |  |
|  | 1 | 16 (32) | 33 (66) | 0 (0) | 0 (0) | 1 (2) | 14 (29) | 34 (69) | 0 (0) | 0 (0) | 1 (2) | 11 (22) | 27 (55) | 7 (14) | 2 (4) | 2 (4) | 0.026 |
|  | 2 | 16 (32) | 33 (66) | 0 (0) | 0 (0) | 1 (2) | 14 (29) | 34 (69) | 0 (0) | 0 (0) | 1 (2) | 11 (22) | 27 (55) | 7 (14) | 2 (4) | 2 (4) | 0.026 |
|  | 3 | 13 (26) | 37 (74) | 0 (0) | 0 (0) | 0 (0) | 18 (37) | 27 (55) | 4 (8) | 0 (0) | 0 (0) | 1 (2) | 35 (71) | 7 (14) | 2 (4) | 4 (8) | <0.001 |
|  | 4 | 40 (41) | 56 (58) | 0 (0) | 0 (0) | 1 (1) | 0 (0) | 2 (50) | 2 (50) | 0 (0) | 0 (0) | 1 (2) | 36 (77) | 5 (11) | 2 (4) | 3 (6) | <0.001 |

* Lep = lepidic, Aci = acinar, Pap = papillary, MP = micropapillary, Sol = solid subtype, Numbers in parentheses are percentages.
